# Epidemiologic and clinical characteristics of *Mycobacterium tuberculosis* infection in Guangxi, China, 2018-2023: a retrospective study

**DOI:** 10.3389/fcimb.2026.1883155

**Published:** 2026-08-03

**Authors:** Dewu Bi, Chaoyou Chen, Hailing Yu, Qiuying Ma, Xiaocheng Luo, Huawei He, Xike Tang, Chaojuan Liang, Lemin Wen, Shaoyong Xi, Zeduan Liu, Wanhong Huang, Qian Long, Hanzhen Su, Xiaolu Luo, Wei Wei, Zizhi Pang, Ningrong Huang, Zongyou Wei, Honghua Shao, Zhouhua Xie

**Affiliations:** 1 Department of Clinical Laboratory, The Fourth People’s Hospital of Nanning, Nanning, Guangxi, China; 2 Key Laboratory of Infectious Diseases, The Nanning Municipal Health Commission, Nanning, Guangxi, China; 3 Human Immunodeficiency Virus/Acquired Immune Deficiency Syndrome (HIV/AIDS) Clinical Treatment Center of Guangxi, Nanning, Guangxi, China; 4 Department of Medical Informatics and Statistics, The Fourth People’s Hospital of Nanning, Nanning, Guangxi, China; 5 Research Laboratory of Infectious Diseases, The Fourth People’s Hospital of Nanning, Nanning, Guangxi, China; 6 Department of Infectious Diseases, The Fourth People’s Hospital of Nanning, Nanning, Guangxi, China

**Keywords:** clinical characteristics, drug resistance, epidemiology, risk factors, tuberculosis

## Abstract

**Background:**

Tuberculosis, caused by *Mycobacterium tuberculosis*, continues to be one of the leading causes of death globally. We performed a retrospective cohort study in which individuals with suspected tuberculosis were evaluated the differences between tuberculosis and other clinically ill patients.

**Methods:**

This study retrospectively analyzed patients with suspected tuberculosis who were hospitalized for pneumonia between January 2018 and December 2023 based on an electronic medical records database.

**Results:**

37,204 patients were included in this study, comprising 34,429 patients with tuberculosis and 2,775 patients with non-tuberculosis lung diseases. Male, human immunodeficiency virus, hepatitis B virus, cytomegalovirus, diabetes, and stroke are risk factors associated with an increased infection of tuberculosis. Poor adherence and human immunodeficiency virus infection are linked to the development of antibiotic resistance in *Mycobacterium tuberculosis*. Resistance to first-line antibiotics usually appears before second-line treatments, with higher resistance rates for first-line antituberculous drugs than for second-line drugs. Isoniazid resistance developed before rifampicin resistance. The degree of bacterial coinfection among patients with tuberculosis is diverse.

**Conclusion:**

This study identified risk factors accounting for the infection and drug resistance of tuberculosis in Guangxi, China, from other illness. The diversity of coinfections among patients with tuberculosis highlights the importance of raising awareness about the multiplicity of infections.

## Introduction

*Mycobacterium tuberculosis* (*M. tuberculosis*) is the bacterium responsible for tuberculosis, a critical public health concern that jeopardizes human health and life (D. Liu et al., 2024; Sousa et al., 2020). The latest estimate from the World Health Organization revealed that 10.7 million people were infected with tuberculosis globally in 2024, with 1.08 million resulting in deaths WHO (2025). The persistence of tuberculosis-related deaths, despite the anti-tuberculosis treatment program, highlights the challenges in diagnosing, designing, managing, and monitoring tuberculosis treatment programs.

The likelihood of patients missing out on treatment was linked to certain demographic and clinical factors (Abera et al., 2022). For instance, older individuals were notably less likely to be administered for the essential anti-tuberculosis treatment. Additionally, individuals with negative smear results or who skipped smear tests were also less likely to receive treatment. This implies that vulnerabilities and comorbidities could interfere with the administration of appropriate care, thus increasing the risk of mortality among tuberculosis patients.

The development of drug-resistant *M. tuberculosis* isolates has had a significant negative impact on tuberculosis control. Approximately 390,000 cases of rifampicin-resistant and multidrug-resistant tuberculosis (RR/MDR-TB) were reported worldwide in 2024, with an overall treatment success rate of 68% (WHO, 2025). The global fight against tuberculosis is still heavily impacted by the health and economic challenges posed by MDR-TB.

Poor adherence to medication and lack of access to treatment are the main reasons for *M. tuberculosis* drug resistance (Barozi et al., 2022). The rise and widespread occurrence of multidrug-resistant *M. tuberculosis* (MDR-TB) have significantly impacted the effectiveness of tuberculosis treatment strategies (Chen et al., 2019; Chesov et al., 2021; Franke et al., 2021). MDR-TB has significant clinical consequences because it necessitates the use of second-line medications, which are typically less effective, more harmful, and require longer treatment periods than first-line treatments. This impacts patient outcomes and significantly strains healthcare systems, especially in areas with limited resources. Furthermore, the rise of extensive drug-resistant (XDR) and totally drug-resistant *M. tuberculosis* strains worsens the problem, as these strains resist even more medications, rendering them almost untreatable with existing treatments. Tackling the worldwide MDR-TB crisis necessitates a comprehensive strategy that involves enhancing diagnostic tools, refining treatment plans, and strengthening public health measures to prevent the emergence and transmission of drug-resistant tuberculosis.

China is among the countries with the highest tuberculosis infection rates in the world. An estimated 700,000 new cases of tuberculosis and nearly 30,000 cases of rifampicin-resistant and multidrug-resistant tuberculosis are diagnosed annually (WHO, 2024). The spatial distribution of tuberculosis in China is characterized by significant regional inequalities, with western regions experiencing higher notification rates than the eastern and central regions (Mao et al., 2020). The gap emphasizes the importance of focused interventions and advancements in economic and medical services to improve tuberculosis case detection and decrease tuberculosis risk throughout the country. In China, the population migrating from rural to urban areas is at high risk for tuberculosis infection and spread, facing obstacles such as financial limitations, insufficient referrals to tuberculosis clinics, and possible social stigma. Addressing these challenges is key to advancing tuberculosis control outcomes in China.

As tuberculosis control measures and various influencing factors are put into place, the epidemiological features of tuberculosis are gradually transforming (Jiang et al., 2024). Throughout history, tuberculosis has been a major public health concern, strongly connected to socio-economic conditions such as poverty, poor nutrition, crowded living conditions, and compromised immunity. Since Koch’s identification of its infectious nature in 1882, there have been substantial advancements in comprehending the history and pathophysiology of tuberculosis. Nevertheless, tuberculosis remains a persistent global health menace, with millions of new cases and fatalities reported each year. Human immunodeficiency virus (HIV) is a risk factor for tuberculosis and has played a major role in its resurgence (Cords et al., 2021; Fojo et al., 2017; Zürcher et al., 2021). Additionally, it contributes to the resistance of *M. tuberculosis* to rifampicin (Cox et al., 2021). In light of the high incidence of *M. tuberculosis* infection and the fact that China is one of the high tuberculosis burden countries, we aim to characterize the epidemiology of tuberculosis in Guangxi, China.

## Materials and methods

### Study setting

This study was conducted at a specialized hospital for infectious diseases. An observational, retrospective cohort study was conducted on inpatient hospitalizations. The inclusion criteria were as follows: (a) patients with respiratory symptoms (wheezing, cough, shortness of breath, and chest tightness) or pulmonary radiographic changes; (b) patients with pyrexia of unknown origin, night sweats, and weight loss; (c) hospitalizations, with a follow-up interview conducted at least 8 weeks after discharge; and (d) all patients with suspected *M. tuberculosis* should receive examinations for acid-fast bacilli smear, culture, or nucleic acid testing. Patients with missing data due to lost follow-up were excluded, along with outpatients.

### Patients

All patients admitted between January 1, 2018, and December 31, 2023, with suspected tuberculosis were retrospectively included in this study, which aimed to assess the epidemiological features and clinical characteristics of tuberculosis among individuals presenting with symptoms indicative of the disease. Suspected tuberculosis is defined as a condition in which patients often present with tuberculosis-like symptoms, such as fever, cough, and shortness of breath lasting more than two weeks, frequently accompanied by imaging changes observed in chest X-rays or CT scans. Regular clinical and laboratory results were continuously documented during admission. Non-tuberculosis patients were defined as those with respiratory diseases other than tuberculosis who were able to expect sputum samples.

Clinical samples, including sputum, bronchoalveolar lavage fluid, feces, pus, urine, and blood, were obtained. Each sample was used for molecular and conventional microbiologic testing, which included concentrated acid-fast bacilli smear microscopy, culture of *Mycobacterium tuberculosis*, and nucleic acid detection. The smears were analyzed for acid-fast bacilli via auramine O fluorescent stain (BaSO, Guangdong, China). The tubes were placed in the BD BACTEC MGIT 960 Mycobacterium Detection System (Becton, Dickinson, Sparks, USA) and incubated at 37 °C for no more than 42 days. *M. tuberculosis* was identified via matrix-assisted laser desorption ionization-time-of-flight mass spectrometry (MALDI-TOF MS) (BRUKER, Massachusetts, USA) following positive culture. The procedures used for molecular diagnosis of *M. tuberculosis*, such as GeneXpert MTB/RIF (Cepheid, Sunnyvale, CA, USA), post-PCR melt curve analysis for antibiotic resistance genes (ZEESAN BIOTECH, Xiamen, China), and mycobacterium species identification (ZEESAN BIOTECH, Xiamen, China), have been described previously (Bi et al., 2023). Acquired drug resistance, also known as drug resistance, occurs when mutations enable cells to withstand treatment. Tuberculosis was confirmed by the presence of a ‘TB detected result’ in the laboratory register or a documented TB diagnosis in patient records.

After the patient is discharged, they will be followed up every two weeks for three consecutive months. After that, follow-up appointments will occur once a month. Collect sputum or lavage fluid for tuberculosis smear, culture, and drug resistance gene monitoring during each follow-up visit. Testing for drug susceptibility on all *Mycobacterium tuberculosis* isolates was conducted using the minimum inhibitory concentration and minimum bactericidal concentration methods.

### Statistical analysis

A Mann-Whitney test was used to compare the medians, whereas *t*-tests were used to compare the means of normally distributed data. Chi-square testing was performed to assess categorical variables. Logistic regression was employed to calculate odds ratios (ORs) and 95% confidence intervals (CIs), which reflect the differences between tuberculosis and other clinically ill patients. The incidence rate ratio (IRR) of occurrences, coupled with the 95% CI, was calculated via Poisson regression. All analyses were performed with MedCalc^®^ (version 15.8, Ostend, Belgium) and GraphPad Prism (version 9.3, California, USA). *P*-values less than 0.05 were considered statistically significant.

## Results

### Epidemiological features of tuberculosis

**Figure 1:**
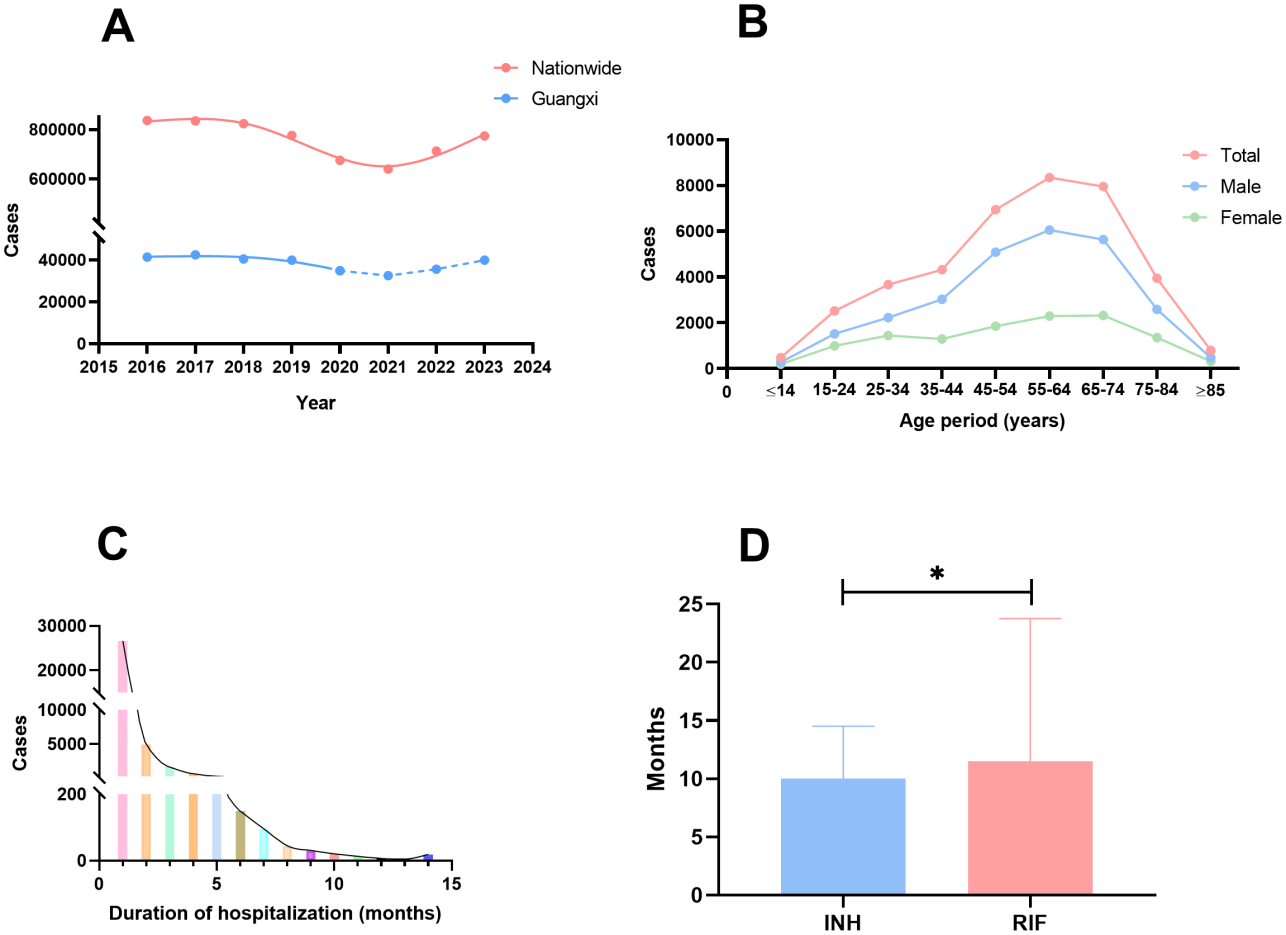
Epidemiological and clinical characteristics of tuberculosis in Guangxi, China, 2018-2023. **(A)** The epidemiological characteristics of tuberculosis have shown dynamic changes in Guangxi, China, from 2018 to 2023. **(B)** Dynamic changes in tuberculosis cases from 2018–2023 were analyzed by age and/or sex, revealing an initial spike followed by a decline in Guangxi, China. **(C)** The length of stay for the 34,429 tuberculosis patients admitted to the hospital during the study was approximately one month for most patients. **(D)** Resistance to isoniazid appeared in *Mycobacterium tuberculosis* prior to rifampicin resistance.

**Figure 2:**
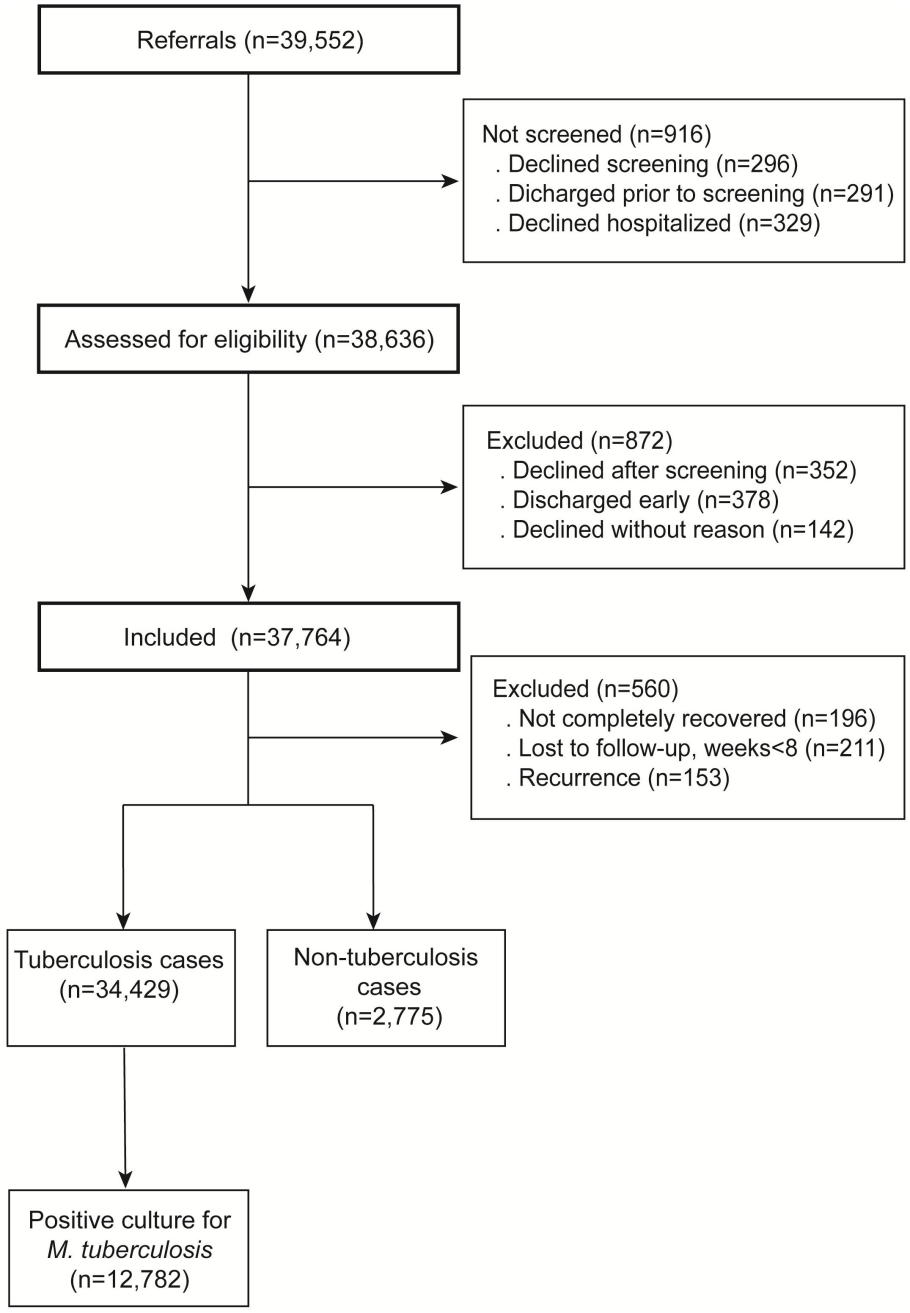
Participant flow diagram.

According to the base-case scenario, the annual number of tuberculosis cases in Guangxi and China between 2016 and 2023 is shown in **Figure 1A**. A decrease in tuberculosis reported cases was observed from 2016-2021, but an increase was observed from 2022. This study included 37,204 cases, comprising 34,429 tuberculosis cases and 2,775 patients with non-tuberculosis lung diseases, without missing variables, identified from a total of 39,552 cases (**Figure 2**). The demographics and clinical characteristics of tuberculosis and non- tuberculosis patients are summarized in **Table 1**. Notable differences in the baseline characteristics of tuberculosis between men and women were observed, with men becoming particularly susceptible to the disease (OR = 1.2639, [95% CI 1.1659-1.3703], *P* < 0.0001). Two peak ages of onset were observed in women: one between the ages of 25 and 34 years and the other between 65 and 74 years; however, an apparent pattern was evident in tuberculosis number, with a peak among men aged 55 to 64 years (**Figure 1B**).

**Table 1:** Characteristics of controls and cases.

Variable	Non-tuberculosis cases (2775) (n=2775)	Tuberculosis cases (n=34429)	*P*-value
Gender			
Male	1773 (64%)	23783 (69%)	0.0015
Female	1002 (36%)	10646 (31%)	
Age (years)			
≤14	54 (1.9%)	285 (0.9%)	<0.0001
15-24	129 (4.6%)	2296 (6.7%)	0.0001
25-34	181 (6.5%)	3272 (9.5%)	<0.0001
35-44	260 (9.4%)	3831 (11.1%)	0.0072
45-54	513 (18.5%)	6074 (17.6%)	0.3092
55-64	660 (23.8%)	7310 (21.2%)	0.0052
65-74	608 (21.9%)	7121 (20.7%)	0.1726
75-84	309 (11.1%)	3542 (10.3%)	0.1820
≥85	61 (2.2%)	698 (2.0%)	0.5444
Occupations			
Farmer	1595 (57.48%)	19650 (57.07%)	0.7868
Unemployed	237 (8.54%)	3334 (9.68%)	0.8019
Freelance	100 (3.60%)	1575 (4.57%)	0.0204
Students	117 (4.22%)	1139 (3.31%)	0.0123
Self-employed	25 (0.90%)	421 (1.22%)	0.1363
Clerks	43 (1.55%)	707 (2.05%)	0.0721
Civil servants	1 (0.04%)	78 (0.23%)	0.0362
Teacher	10 (0.36%)	154 (0.45%)	0.5070
Worker	26 (0.94%)	390 (1.13%)	0.3480
Fishers	2 (0.07%)	27 (0.08%)	0.9082
Unknown	288 (10.38%)	3288 (9.55%)	0.1758
Medical staff	6 (0.22%)	70 (0.20%)	0.8850
Children	10 (0.36%)	85 (0.25%)	0.2551
Retirees	221 (7.96%)	2654 (7.71%)	0.6416
Others	94 (3.39%)	857 (2.49%)	0.0044
Comorbidities *			
HIV	199 (7.17%)	3550 (10.31%)	<0.0001
HBV	132 (4.76%)	2423 (7.04%)	<0.0001
HCV	23 (0.83%)	397 (1.15%)	0.1220
CMV	15 (0.54%)	511 (1.48%)	0.0001
COVID-19	42 (1.51%)	436 (1.27%)	0.2692
Clonorchis sinensis	148 (5.33%)	1922 (5.58%)	0.5924
Cryptococcosis	17 (0.61%)	126 (0.37%)	0.0438
Talaromyces marneffei	66 (2.38%)	584 (1.70%)	0.0089
Syphilis	21 (0.76%)	423 (1.23%)	0.0286
Aspergillosis	24 (0.86%)	439 (1.28%)	0.0624
Pneumocystis pneumonia	40 (1.44%)	357 (1.04%)	0.0472
Autoimmune diseases	163 (5.87%)	1586 (4.61%)	0.0031
Diabetes mellitus	246 (8.86%)	4759 (13.82%)	<0.0001
Tumor	791 (28.5%)	2783 (8.08%)	<0.0001
Stroke	180 (6.49%)	3068 (8.91%)	<0.0001
Hypertension	492 (17.73%)	5887 (17.10%)	0.4401
Urinary calculi	94 (3.39%)	1344 (3.90%)	0.1833

*Some patients had multiple comorbidities.

Data are presented as count (%).

*P* values are the significance level of the chi square test.

The number of tuberculosis cases increased with age (**Table 1**). The duration of hospitalization associated with tuberculosis is lengthy (**Figure 1C**). As a result of pneumonia, more than half of the tuberculosis patients were hospitalized for one month and required antituberculosis treatment.

Variables affecting the disparities between tuberculosis and other sick patients

Individuals infected with HIV are more susceptible to *M. tuberculosis* infection than are those without HIV (OR = 1.5502, [95% CI 1.3424-1.7903], *P* < 0.0001). Among patients with cytomegalovirus infection, the odds ratio for infection in those with tuberculosis was 2.7721 (95% CI 1.6565-4.6391, *P* = 0.0001). Infection with hepatitis B virus (HBV) is associated with an increased risk of tuberculosis (OR = 1.5158, [95% CI 1.2666-1.8141], *P* < 0.0001). Nevertheless, infection with the hepatitis C virus had no significant effect on tuberculosis at onset. The odds ratio for the development of tuberculosis in diabetic patients was 1.6490 (95% CI 1.4415-1.8862, *P* < 0.0001). No significant impact of hypertension on tuberculosis at onset was observed. Compared with patients without stroke, the odds ratio for the development of tuberculosis in patients with stroke was 1.4104 (95% CI 1.2072-1.6477, *P* < 0.0001).

### Microbial profile

**Figure 3:**
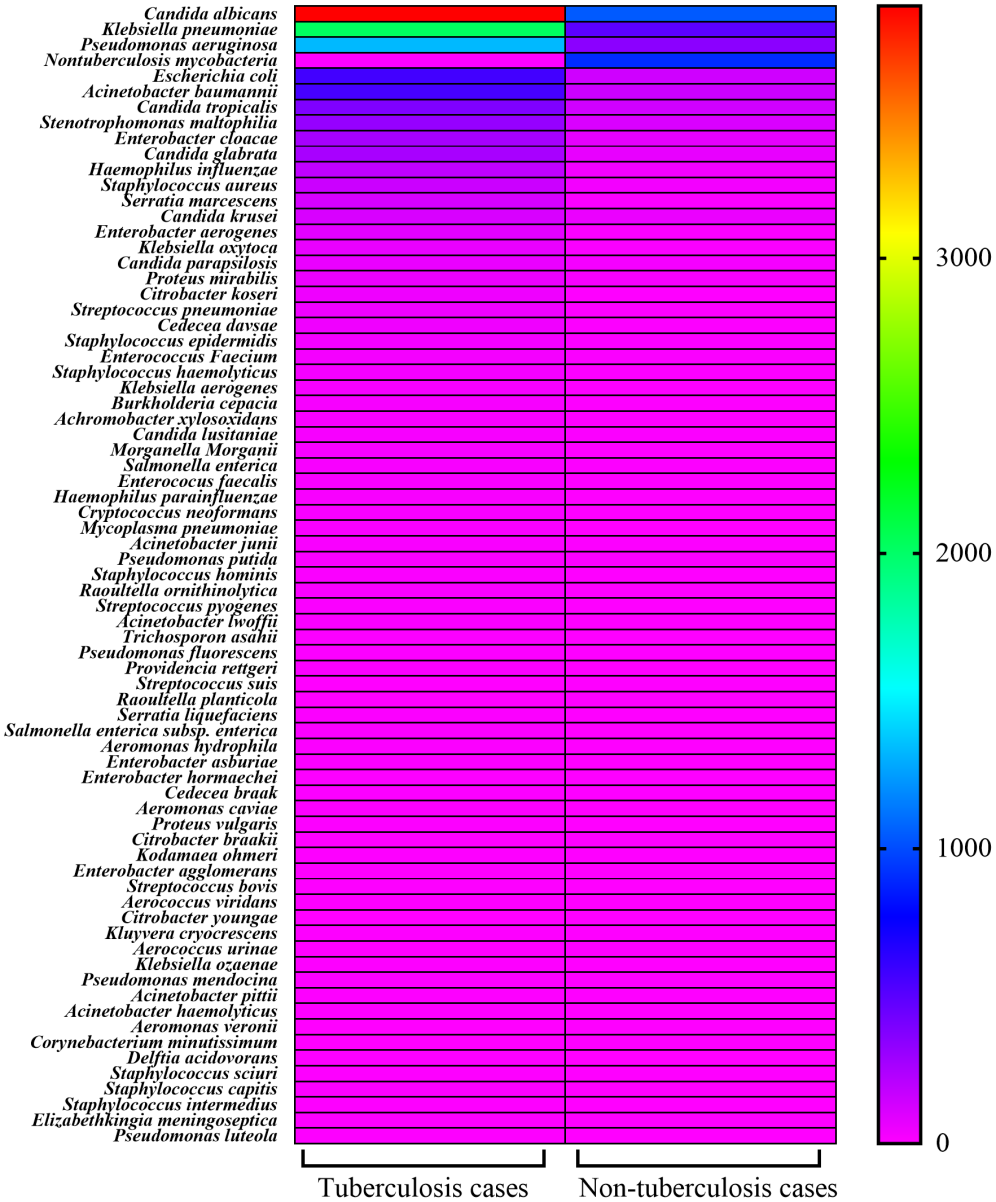
Distribution of confirmed laboratory pathogens coexisting with infections among hospitalized tuberculosis patients in Guangxi, China, between 2018 and 2023. The biodiversity of pathogenic microorganisms was assessed in samples collected from both tuberculosis and non-tuberculosis cases.

The microbial profile observed varied significantly between the tuberculosis and non- tuberculosis patients (**Figure 3**). Microbial diversity was greater in tuberculosis cases than in non-tuberculosis patients (OR = 3.3496, [95% CI 2.3966-4.6816], *P* < 0.0001), whereas tuberculosis patients presented lower infection rates for each pathogen (**Supplementary Table 4**).

### Drug resistance

The Drug Susceptibility Test Kit for Mycobacteria (Culture Method) identified anti-TB drug-resistant strains, which were further analyzed for antibiotic-resistant genes using the MeltPro^®^ MTB/INH, RIF, and FQ Test Kit (MMCA) from ZEESAN BIOTECH, Xiamen, China. Out of 3,779 drug-resistant *M. tuberculosis* isolates, 1,205 were identified as multidrug-resistant clinical isolates. PCR and melting curve analysis methods were utilized to investigate various gene classes linked to antibiotic resistance. Specifically, the kit employed PCR and melting curve analysis to identify mutations in the *rpoB* gene (codons 507-533) for rifampin resistance, the *gyrA* gene (sites 88~94) for Fluoroquinolone resistance, and the *ahpC* promoter region (positions -44~-30 and -15~3), *inhA* codon 94, *inhA* promoter region (positions -17~-8), and *katG* codon 315 for Isoniazid resistance determination. The most commonly mutated sites were the RNA polymerase subunit *rpoB* gene (codons 507-533) and *katG* codon 315, detected in 28.2% (340/1205) of the multidrug-resistant clinical isolates in this study. No mutations in the coding regions were found within the amplicons covering the mutation sites in 0.5% (6/1205) of the multidrug-resistant clinical isolates.

Drug resistance is common because medications for *M. tuberculosis* are usually recommended for use in conjunctions. Antibiotic resistance to first-line medications typically emerges before resistance develops to second-line treatments, and the resistance rates of first-line antituberculous drugs often surpass those of second-line drugs. The infection of multidrug-resistant tuberculosis (MDR-TB) was the highest, with a peak of approximately 10% (**Supplementary Table 1**). The infection of isoniazid resistance was greater than that of rifampicin resistance, although rifampicin, alongside isoniazid, is a fundamental component of the initial treatment of drug-sensitive tuberculosis. Significant differences were observed in the time it took for antituberculosis drugs to induce drug-resistant tuberculosis (**Supplementary Table 3**). Compared with rifampicin, the time required for isoniazid resistance to develop in *M. tuberculosis* was significantly shorter during the treatment of drug-sensitive tuberculosis (10 months [IQR: 4-15] *vs* 12 months [IQR: 8-24], *P* = 0.0318) (**Figure 1D**). Poor adherence was linked to drug resistance; the odds ratio for the development of drug-resistant tuberculosis in patients with drug-sensitive tuberculosis was 3.1043 (95% CI: 1.455-6.621, *P* = 0.0034). Moreover, HIV acquisition was found to be associated with an increased risk of drug-resistant tuberculosis infection (OR = 1.4025 [95% CI: 1.0172-1.9337], *P* = 0.0390). The mutations in *katG* 315 and *rpoB* 529–533 were significantly associated with isoniazid and rifampicin resistance, respectively (**Supplementary Table 2**). Interestingly, mutations in resistance genes were not detected in any of the multidrug-resistant tuberculosis isolates.

The odds ratio of developing multidrug-resistant tuberculosis was greater in men (OR = 2.7683 [95% CI: 2.2428-3.4170], *P* < 0.0001). The risk of multidrug-resistant tuberculosis is significantly greater in men aged 45 years or older than in men aged younger than 45 years (OR = 1.2700 [95% CI: 1.0127-1.5927], *P* = 0.0385). The opposite was true for women (OR = 1.4848 [95% CI: 1.0156-2.1709], *P* = 0.0413).

## Discussion

In this study, we report the epidemiological and clinical characteristics of *M. tuberculosis* infection in Guangxi, China, from 2018–2023 and investigate risk factors associated with tuberculosis. Consistent with previous studies of tuberculosis epidemiology (Zhou et al., 2016; Meintjes et al., 2019), we found that older men, HIV status, and diabetes are significantly associated with an increased risk of tuberculosis infection. Our current study revealed that isoniazid resistance precedes rifampicin resistance, and the most common mutation sites are *katG* at position 315 and *rpoB* at positions 529–533 in the multidrug-resistant forms of *M. tuberculosis*, which are known as hotspot mutations. However, self-reported age at tuberculosis onset in women presents a bimodal pattern, with the first peak occurring during adolescence and the second occurring in older individuals. Furthermore, HBV is a risk factor contributing to the increasing infection of tuberculosis. Our research indicates that HIV not only influences the incidence of tuberculosis but also contributes to the development of drug resistance. The results of our study imply that *M. tuberculosis* frequently coinfects other pathogens and is diverse.

Prior studies demonstrated that pregnant women with both HIV and tuberculosis had poorer outcomes for themselves and their infants, indicating that the dual burden of these conditions can lead to increased vulnerability at specific life stages (Dooley et al., 2014; Matthews et al., 2013). Various epidemiological and immunological factors that influence the infection and progression of tuberculosis can explain the observation of two peaks in infected women. The infection of tuberculosis tends to increase with age, highlighting the need for targeted screening and prevention efforts in older populations.

Our results are in agreement with those of prior studies (Ebrahimzadeh et al., 2023; van Deursen et al., 2022); a significant decrease in tuberculosis infection was observed in all populations in Guangxi, China, during the COVID-19 pandemic. The decrease in tuberculosis cases may be due to the adoption of improved personal protective strategies, timely diagnosis, and the use of sufficient doses of antituberculosis drugs, along with monitoring for patient compliance.

Individuals living with HIV, even those with higher CD4 levels, are more likely to contract tuberculosis due to the immunosuppressive nature of HIV, which might still prevent a similar response to treatment (Franke et al., 2021). The dynamics of coevolution between HIV and *M. tuberculosis* may differ in patients who are coinfected with both HIV and tuberculosis (Koch et al., 2017). Therefore, the correlation between HIV and the evolution of drug-resistant *M. tuberculosis* has rarely been reported. A previous study reported that HIV is associated with the development of rifampicin resistance during first-line treatment for tuberculosis (Cox et al., 2021). In this study, we employed a combination approach to assess the development of drug resistance in *M. tuberculosis* comprehensively among patients with both HIV and tuberculosis. Our investigation suggested that HIV may contribute to the development of drug resistance during long-term tuberculosis treatment, which might cause a rise in multidrug-resistant and extensive drug‐resistant tuberculosis over time. Cytomegalovirus is the predominant infection in individuals with AIDS. Therefore, we observed that cytomegalovirus was a significant risk factor for tuberculosis.

Interestingly, our study suggests that the hepatitis B virus contributes to an increased risk of tuberculosis. Tuberculosis and hepatitis B virus infection are prevalent at high rates in China (Liu D. et al., 2024; Liu R. et al., 2024). More than 80% of hepatocellular carcinoma cases are attributed to infection with the hepatitis B virus (Chen et al., 2021; Feng et al., 2023). Patients with resolved hepatitis B virus infection who are immunosuppressed face a heightened risk of hepatitis B virus reactivation, which can lead to severe liver issues, cirrhosis, hepatitis B virus-related liver failure, and potentially death related to hepatitis B virus (Yin et al., 2023). The immune system is considered to be suppressed by tumor chemotherapy, which is associated with an increased risk of infection, especially tuberculosis (Lin et al., 2023).

Increasing evidence suggests that pulmonary tuberculosis may increase susceptibility to infections by other pathogenic bacteria (Buchera et al., 2022). Pulmonary tuberculosis not only directly endangers individuals through mycobacterial infection but also heightens the likelihood of secondary bacterial infections in the respiratory tract. This heightened risk is a consequence of lung tissue damage, immune system dysregulation, and potential genetic predispositions. Comprehending these interactions is vital for formulating holistic treatment approaches that target both tuberculosis and the susceptibility to secondary infections, thereby enhancing patient prognosis.

Approximately 1% of the multidrug-resistant tuberculosis isolates presented no mutations in mutation hotspots associated with isoniazid- and rifampicin resistance genes. Our analysis suggests that the results of this study may reveal previously unknown mechanisms of medication resistance. Whole-genome sequencing research is needed for more precise mapping of these potential regions or mutations.

Genetic mutations that lead to resistance against primary anti-tuberculosis drugs add to the global challenge of MDR-TB. Studies have identified that the S315T mutation in the *katG* gene and the S531L mutation in the *rpoB* gene are prevalent mutations linked to MDR-TB, occurring in various geographic areas (Hossain et al., 2026; Motavaf et al., 2021). Research in Casablanca, Morocco, demonstrated a notable prevalence of fluoroquinolone resistance, with mutations in the *gyrA* and *gyrB* genes being predominant among MDR-TB isolates (Oudghiri et al., 2018). In Southern Brazil, genomic analysis uncovered a high transmission rate of MDR-TB, with a notable portion of strains attributed to Lineage 4 (Salvato et al., 2021). This underscores the significance of genomic surveillance in pinpointing transmission hotspots and guiding public health interventions. Additionally, research from Nepal indicated that the emergence of second-line drug resistance is likely indigenous rather than stemming from the introduction of resistant strains, underscoring the importance of local tuberculosis control programs in addressing drug resistance (Leong et al., 2022). The advent of rapid molecular diagnostic assays, like the real-time-PCR-based melting curve analysis, has transformed the detection of drug-resistant TB (Hu et al., 2014). These assays exhibit high sensitivity and specificity in identifying resistance to rifampicin and isoniazid, facilitating prompt diagnosis and management of MDR-TB cases.

Infection with *M. tuberculosis*, which causes tuberculosis, frequently occurs via the respiratory tract. Inflammation in the respiratory tract and lungs leads to changes in airway inflammation and lung function, both physiologically and pathophysiologically. Opportunistic infections are common in patients with tuberculosis throughout the illness. Opportunistic pathogens are characterized by microbial diversity.

In agreement with the findings of previous studies (Zhou et al., 2016; Lee et al., 2016), diabetes, which is known to increase the risk of tuberculosis, has a significant impact on the infection of this disease. Clonorchiasis is a food-borne parasitic condition caused by Clonorchis sinensis and is predominantly endemic in Guangxi, China (Kan et al., 2022). However, our study revealed that endemic clonorchiasis had no noticeable effect on the infection of tuberculosis.

We acknowledge the following limitations in the study: single-center study, referral bias, retrospective design, and absence of smoking data. As a retrospective analysis, the current study has the typical limitations associated with this type of research. Additional investigations featuring whole-genome sequencing are needed to identify potential mutation locations or genes in antibiotic-resistant isolates of *M. tuberculosis*.

## Data Availability

The original contributions presented in the study are included in the article/**Supplementary Material**. Further inquiries can be directed to the corresponding authors.
