# Supplementary material for: Epidemiologic and clinical characteristics of Mycobacterium tuberculosis infection in Guangxi, China, 2018-2023: a retrospective study
Source: Front Cell Infect Microbiol. 2026 Aug 3;16:1883155. doi: 10.3389/fcimb.2026.1883155 (PMC13478008; doi:10.3389/fcimb.2026.1883155)
Supplement: Supplementary file 1 [file SupplementaryFile1.docx]

**Table S1. Infection of drug-resistant tuberculosis in Guangxi, China, 2018-2023.**

|  | 2018 | 2019 | 2020 | 2021 | 2022 | 2023 |
| --- | --- | --- | --- | --- | --- | --- |
| HR-TB | 115 (5.5%) | 128 (5.3%) | 146 (7.0%) | 113 (9.2%) | 195 (6.8%) | 130 (7.6%) |
| RR-TB | 70 (3.3%) | 54 (2.2%) | 34 (1.6%) | 26 (2.1%) | 160 (5.6%) | 32 (1.9%) |
| PDR | 74 (3.5%) | 77 (3.2%) | 108 (5.2%) | 64 (5.2%) | 93 (3.3%) | 74 (4.3%) |
| MDR | 228 (10.8%) | 250 (10.4%) | 225 (10.7%) | 124 (10.1%) | 245 (8.6%) | 125 (7.3%) |
| XDR | 103 (4.9%) | 85 (3.5%) | 62 (3.0%) | 14 (1.1%) | 65 (2.3%) | 26 (1.5%) |

HR-TB, isoniazid-resistant tuberculosis

RR-TB, rifampicin-resistant tuberculosis

PDR, poly drug-resistant tuberculosis

MDR, multidrug-resistant tuberculosis

XDR, extensively drug-resistant tuberculosis

**Table S2. Distribution of mutations in drug-resistant *M. tuberculosis* clinical isolates.**

| Drug resistance mutations and sites | Percentage (%) |
| --- | --- |
| *katG315*; *rpoB529～533* | 28.2% |
| Rifampin-susceptible; *katG315* | 14.1% |
| Isoniazid-susceptible; *rpoB529～533* | 12.1% |
| *katG315*; *rpoB521～528* | 5.8% |
| *katG315*; *rpoB507～512* | 3.4% |
| *katG315*; *rpoB513～520*, *rpoB521～528* | 3.4% |
| Isoniazid-susceptible; *rpoB521～528* | 2.4% |
| *inhA-17～-8*; *rpoB529～533* | 2.4% |
| *katG315*; *rpoB533* | 2.4% |
| Rifampin-susceptible; *inhA-17～-8* | 1.9% |
| *inhA-17～-8*; *rpoB529～533* | 1.9% |
| *inhA-17～-8*; *katG315*; *rpoB529～533* | 1.9% |
| *katG315*; *rpoB507～512*, *rpoB513～520* | 1.5% |
| *katG315*; *rpoB507～512*, *rpoB521～528* | 1.5% |
| *katG315*; *rpoB513～520*, *rpoB521～528*, *rpoB529～533* | 1.5% |
| *ahpC-44～-30*, *ahpC-15～3*; *rpoB513～520* | 1.0% |
| Isoniazid-susceptible; *rpoB513～520*, *rpoB521～528* | 1.0% |
| Isoniazid-susceptible; *rpoB521～528*, *rpoB529～533* | 1.0% |
| Rifampin-susceptible; *inhA-17～-8*; *katG315* | 1.0% |
| *inhA-17～-8*; *katG315*; *rpoB533* | 1.0% |
| *inhA94*; *katG315*; *rpoB529～533* | 1.0% |
| *katG315*; *rpoB513～520*; *rpoB529～533* | 1.0% |
| *ahpC-44～-30*, *ahpC-15～3*; *rpoB529～533* | 0.6% |
| *ahpC-44～-30*, *ahpC-15～3*; *rpoB529～533*; *katG* deletion | 0.5% |
| *ahpC-44～-30*, *ahpC-15～3*; *rpoB507～512*, *rpoB513～520* | 0.5% |
| *ahpC-44～-30*, *ahpC-15～3*; *rpoB507～512*, *rpoB513～520* | 0.5% |
| *ahpC-44～-30*, *ahpC-15～3*; *rpoB529～533*; *katG315* | 0.5% |
| Isoniazid-susceptible; *rpoB513～520* | 0.5% |
| Isoniazid-susceptible; *rpoB513～520*, *rpoB529～533* | 0.5% |
| Isoniazid-susceptible; *rpoB533* | 0.5% |
| Rifampin-susceptible; *ahpC-44～-30*, *ahpC-15～3*; *inhA-17～-8* | 0.5% |
| *inhA-17～-8*; *rpoB513～520*, *rpoB521～528* | 0.5% |
| *inhA-17～-8*; *katG315*; *rpoB521～528* | 0.5% |
| *inhA-17～-8*; *katG315*; *rpoB513～520* | 0.5% |
| Isoniazid and Rifampin-susceptible | 0.5% |
| Rifampin-susceptible; *inhA-17～-8*, *inhA94* | 0.4% |
| *inhA-44～-30*; *rpoB521～528* | 0.4% |
| *inhA94*; *katG315*; *rpoB513～520*, *rpoB521～528* | 0.4% |
| *katG315*; *rpoB529～533* | 0.4% |
| *katG315*; *rpoB521～528*, *rpoB529～533* | 0.4% |

**Table S3. Length of time required to result in drug-resistant *M. tuberculosis.***

| Antitubercular drugs | Time, months (median, IQR) | 95% CI of median |
| --- | --- | --- |
| Streptomycin | 12 (9-25) | 9-26 |
| Ethambutol | 11 (6-17) | 7-16 |
| Isoniazid | 10 (4-15) | 6-12 |
| Rifampicin | 12 (8-24) | 9-21 |
| Para-aminosalicylic acid | 14 (4-21) | 8-18 |
| Levofloxacin | 15 (10-25) | 12-21 |
| Capreomycin | 5 (2-22) | 1-27 |
| Protionamide | 20 (10-31) | 5-34 |
| Amikacin | 10 (6-21) | 6-21 |
| Rifapentine | 16 (7-24) | 11-23 |
| Pasiniazid | 13 (6-26) | 6-24 |
| Moxifloxacin | 11 (3-26) | 3-25 |
| Clarithromycin | 12 (3-19) | 3-20 |
| Rifabutin | 13 (3-27) | 3-23 |
| Clofazimine | 9 (3-23) | 3-23 |

Analysis of variance (ANOVA) was employed for statistical analysis. Analysis of variance revealed that the length of time required to develop drug-resistant *M. tuberculosis* was significantly different (*P*=0.0075).

Table S4. Major pathogen spectrum differed between cases and controls.

| Pathogens | Tuberculosis cases (34429) | Non-tuberculosis cases (2775) | Incidence rate ratio (95% CI) | *P* value |
| --- | --- | --- | --- | --- |
| *Candida albicans* | 3855 (11.20%) | 1043 (37.59%) | 0.2979 (0.2781-0.3193) | <0.0001 |
| *Klebsiella pneumoniae* | 2022 (5.87%) | 491 (17.69%) | 0.3319 (0.3006-0.3671) | <0.0001 |
| *Pseudomonas aeruginosa* | 1328 (3.86%) | 349 (12.58%) | 0.3067 (0.2724-0.3461) | <0.0001 |
| *Escherichia coli* | 570 (1.66%) | 144 (5.19%) | 0.3190 (0.2653-0.3858) | <0.0001 |
| *Acinetobacter baumannii* | 557 (1.62%) | 153 (5.51%) | 0.2934 (0.2449-0.3533) | <0.0001 |
| *Candida tropicalis* | 385 (1.12%) | 142 (5.12%) | 0.2185 (0.1798-0.2668) | <0.0001 |
| *Stenotrophomonas maltophilia* | 316 (0.92%) | 109 (3.93%) | 0.2337 (0.1874-0.2932) | <0.0001 |
| *Enterobacter cloacae* | 264 (0.77%) | 75 (2.70%) | 0.2837 (0.2188-0.3717) | <0.0001 |
| *Candida glabrata* | 262 (0.76%) | 68 (2.45%) | 0.3105 (0.2370-0.4117) | <0.0001 |
| *Haemophilus influenzae* | 188 (0.55%) | 35 (1.26%) | 0.4329 (0.3006-0.6402) | <0.0001 |
| *Staphylococcus aureus* | 157 (0.46%) | 36 (1.30%) | 0.3515 (0.2434-0.5200) | <0.0001 |
| *Serratia marcescens* | 119 (0.35%) | 9 (0.32%) | 1.0657 (0.5429-2.3878) | 0.8539 |
| *Candida krusei* | 116 (0.34%) | 59 (2.13%) | 0.1585 (0.1149-0.2206) | <0.0001 |
| *Enterobacter aerogenes* | 83 (0.24%) | 9 (0.32%) | 0.7433 (0.3732-1.6825) | 0.3962 |
| *Klebsiella oxytoca* | 66 (0.19%) | 13 (0.47%) | 0.4092 (0.2236-0.8087) | 0.0023 |
| *Candida parapsilosis* | 62 (0.18%) | 28 (1.01%) | 0.1785 (0.1125-0.2897) | <0.0001 |
| *Proteus mirabilis* | 59 (0.17%) | 23 (0.83%) | 0.2068 (0.1258-0.3508) | <0.0001 |
| *Citrobacter koseri* | 47 (0.14%) | 2 (0.07%) | 1.8941 (0.4960-16.0983) | 0.3682 |
| *Streptococcus pneumoniae* | 44 (0.13%) | 5 (0.18%) | 0.7093 (0.2820-2.2920) | 0.4645 |
| *Cedecea davsae* | 43 (0.12%) | 8 (0.29%) | 0.4332 (0.2013-1.0669) | 0.0253 |
| *Staphylococcus epidermidis* | 31 (0.09%) | 6 (0.22%) | 0.4164 (0.1712-1.2210) | 0.0426 |
| *Enterococcus faecium* | 31 (0.09%) | 13 (0.47%) | 0.1922 (0.0977-0.4002) | <0.0001 |
| *Staphylococcus haemolyticus* | 29 (0.08%) | 9 (0.32%) | 0.2597 (0.1197-0.6237) | 0.0001 |
| *Klebsiella aerogenes* | 27 (0.08%) | 9 (0.32%) | 0.2418 (0.1104-0.5844) | 0.0001 |
| *Burkholderia cepacia* | 24 (0.07%) | 5 (0.18%) | 0.3869 (0.1447-1.2982) | 0.0449 |
| *Achromobacter xylosoxidans* | 23 (0.07%) | 3 (0.11%) | 0.6179 (0.1867-3.2149) | 0.4285 |
| *Candida lusitaniae* | 23 (0.07%) | 10 (0.36%) | 0.1854 (0.0848-0.4363) | <0.0001 |
| *Morganella morganii* | 22 (0.06%) | 2 (0.07%) | 0.8866 (0.2180-7.7780) | 0.8705 |
| *Salmonella enterica* | 21 (0.06%) | 7 (0.25%) | 0.2418 (0.0990-0.6733) | 0.0004 |
| *Enterococus faecalis* | 20 (0.06%) | 3 (0.11%) | 0.5373 (0.1594-2.8238) | 0.3080 |
| *Haemophilus parainfluenzae* | 20 (0.06%) | 1 (0.04%) | 1.6120 (0.2578-66.8144) | 0.6381 |
| *Cryptococcus neoformans* | 20 (0.06%) | 4 (0.14%) | 0.4030 (0.1350-1.6215) | 0.0860 |
| *Acinetobacter junii* | 16 (0.05%) | 7 (0.25%) | 0.1842 (0.0717-0.5295) | <0.0001 |
| *Pseudomonas putida* | 15 (0.04%) | 7 (0.25%) | 0.1727 (0.0663-0.5007) | <0.0001 |
| *Staphylococcus hominis* | 15 (0.04%) | 3 (0.11%) | 0.4030 (0.1140-2.1718) | 0.1370 |
| *Raoultella ornithinolytica* | 15 (0.04%) | 5 (0.18%) | 0.2418 (0.0835-0.8504) | 0.0028 |
| *Streptococcus pyogenes* | 15 (0.04%) | 3 (0.11%) | 0.4030 (0.1140-2.1718) | 0.1370 |
| *Acinetobacter lwoffii* | 14 (0.04%) | 3 (0.11%) | 0.3761 (0.1050-2.0413) | 0.1098 |
| *Trichosporon asahii* | 11 (0.03%) | 2 (0.07%) | 0.4433 (0.0967-4.1159) | 0.2767 |
| *Pseudomonas fluorescens* | 11 (0.03%) | 3 (0.11%) | 0.2599 (0.0780-1.6498) | 0.0466 |
| *Serratia liquefaciens* | 11 (0.03%) | 1 (0.04%) | 0.8866 (0.1289-38.1624) | 0.9082 |
| *Aeromonas hydrophila* | 10 (0.03%) | 3 (0.11%) | 0.2687 (0.0692-1.5192) | 0.0321 |
| *Enterobacter asburiae* | 10 (0.03%) | 3 (0.11%) | 0.2687 (0.0692-1.5192) | 0.0321 |
| *Enterobacter hormaechei* | 9 (0.03%) | 2 (0.07%) | 0.3627 (0.0750-3.4497) | 0.1758 |
| *Cedecea braak* | 8 (0.02%) | 4 (0.14%) | 0.1612 (0.0432-0.7315) | 0.0006 |
| *Kodamaea ohmeri* | 6 (0.02%) | 3 (0.11%) | 0.1612 (0.0432-0.7315) | 0.0031 |
| *Aerococcus viridans* | 6 (0.02%) | 1 (0.04%) | 0.4836 (0.0587-22.2446) | 0.4918 |
| *Kluyvera cryocrescens* | 5 (0.01%) | 1 (0.04%) | 0.4030 (0.0451-19.0611) | 0.3906 |

A multivariable logistic regression model adjusting simultaneously for age, sex, HIV, and metabolic comorbidities (MC).

Logistic regression models and odds ratios are as follow:

$P=1/\left\{ 1+Exp \right.\left[ -\left( -1.6788+0.0283Age-0.9931HIV+0.2336MC+0.4624Sex \right) \right]\}$ (Significance level *P*<0.0001)

Odds Ratios and 95% Confidence Intervals

| Variable | Odds ratio | 95% CI | *P* value |
| --- | --- | --- | --- |
| Age | 1.0288 | 1.0281-1.0295 | <0.0001 |
| HIV | 0.3704 | 0.3543-0.3873 | <0.0001 |
| MC | 1.2632 | 1.2043-1.3249 | <0.0001 |
| Sex | 1.5880 | 1.5411-1.6363 | <0.0001 |

HIV acquisition was found to be associated with an increased risk of drug-resistant tuberculosis infection (OR=1.4025 [95% CI: 1.0172-1.9337], *P*=0.0390).

Approximately 37% of the 34,429 tuberculosis cases had a positive culture for *M. tuberculosis*.

| HIV uninfected group  12,620 cases | |  | HIV co-infected group  162 cases | |
| --- | --- | --- | --- | --- |
| Drug-susceptible *M. tuberculosis* | Drug-resistant  *M. tuberculosis* |  | Drug-susceptible *M. tuberculosis* | Drug-resistant  *M. tuberculosis* |
| 8,891 cases | 3,729 cases |  | 102 cases | 60 cases |
